# The relationship of socioeconomic status in childhood and adulthood with compassion: A study with a prospective 32-year follow-up

**DOI:** 10.1371/journal.pone.0248226

**Published:** 2021-03-24

**Authors:** Aino I. Saarinen, Dacher Keltner, Henrik Dobewall, Terho Lehtimäki, Liisa Keltikangas-Järvinen, Mirka Hintsanen

**Affiliations:** 1 Research Unit of Psychology, University of Oulu, Oulu, Finland; 2 Department of Psychology and Logopedics, Faculty of Medicine, University of Helsinki, Helsinki, Finland; 3 Department of Psychology, University of California Berkeley, Berkeley, CA, United States of America; 4 Department of Clinical Chemistry, Fimlab Laboratories and Finnish Cardiovascular Research Center-Tampere, Faculty of Medicine and Health Technology, Tampere University, Tampere, Finland; Montana State University, UNITED STATES

## Abstract

The objective of this study was to investigate (i) whether childhood family SES predicts offspring’s compassion between ages 20–50 years and (ii) whether adulthood SES predicts compassion or vice versa. We used the prospective population-based Young Finns data (*N* = 637–2300). Childhood family SES was evaluated in 1980; participants’ adulthood SES in 2001 and 2011; and compassion for others in 1997, 2001, and 2012. Compassion for others was evaluated with the Compassion scale of the Temperament and Character Inventory. The results showed that high childhood family SES (a composite score of educational level, occupational status, unemployment status, and level of income) predicted offspring’s higher compassion between ages 30–40 years but not in early adulthood or middle age. These results were obtained independently of a variety of potential confounders (disruptive behavior in childhood; parental mental disorder; frequency of parental alcohol use and alcohol intoxication). Moreover, high compassion for others in adulthood (a composite score of educational level, occupational status, and unemployment status) predicted higher adulthood SES later in their life (after a 10-year follow-up), but not vice versa. In conclusion, favorable socioeconomic environment in childhood appears to have a positive effect on offspring’s compassion in their middle adulthood. This effect may attenuate by middle age. High compassion for others seems to promote the achievement of higher SES in adulthood.

## 1 Introduction

There is evidence that children and adolescents coming from socioeconomically disadvantaged circumstances are vulnerable to increased risk for all-cause mortality [1] and even two to three times the risk for mental health problems [2]. Little is known, however, whether low-SES family background predicts offspring’s compassion, even though compassion is strongly linked to one’s physical and mental health.

Compassion refers to a feeling concern for other’s suffering that is followed by the desire to alleviate it [3]. Compassion can take the form of a state, referring to transient compassionate feelings for others that may vary along with situational factors (compassionate states) or to a more stable trait that endures over time and over situations (dispositional compassion) [3]. Compassion-training interventions are shown to effectively promote better mental health by reducing depression, anxiety, stress, and psychotic symptoms [4, 5]. Compassion is a separate construct from empathy: whereas compassion includes a desire to alleviate other’s suffering, empathy refers to an ability to synchronize with others’ emotions [6, 7]. Some degree of empathy is a prerequisite for compassion [8, 9]. Moreover, empathy and compassion relate to partly non-overlapping brain networks [10].

To our knowledge, no longitudinal study has investigated whether childhood family SES might predict offspring’s development of compassion in adulthood. Some researchers have hypothesized that high parental SES might result in more frequent compassionate messages from mother to child [11] as well as teaching the child how to understand others’ emotional states and how to feel concern for others [12]. These parent-child dynamics would suggest that the SES of parents would influence compassionate tendencies in their offspring. Nevertheless, evidence for this is lacking.

To date, select cross-sectional studies have investigated the relationship between parental SES and offspring’s socioemotional qualities in childhood. It has been found that high parental SES is related to children’s higher level of altruistic behavior [13] and higher levels of cognitive and affective empathy in childhood [14, 15]. It has been suggested that this association between high parental SES and children’s higher empathy and altruism may be indirect via other qualities of the family environment. For example, high family SES is proposed to be linked to lower parental stress levels [12, 14], higher maternal support for the offspring [12, 14], higher quality of the parent-child communication [11, 16], and better family climate [12].

Other cross-sectional studies, however, have obtained quite opposite findings about the link between parental SES and offspring’s socioemotional qualities in childhood. Specifically, it has been found that high family SES is related to children’s lower empathy [17] and less frequent altruistic behavior toward others [13, 18]. Findings such as these have led to the speculation that wealthy socioeconomic circumstances might eventually reduce the child’s need for reliance on others [13, 19]. In this way, high family SES in childhood might potentially increase children’s egocentricity and reduce their concern for other’s needs [13, 19].

Taken together, the previous findings have been inconclusive about the link between parental SES and offspring’s socioemotional qualities. Overall, these inconclusive findings may stem from two methodological properties of past studies. Firstly, all the previous studies on this topic have been cross-sectional in design. However, only longitudinal studies with follow-ups into adulthood could shed light on the long-term association between parental SES and offspring’s socioemotional development. Secondly, the previous studies have investigated only single SES indicators (e.g. parents’ level of income or education), which may not have captured the multidimensional nature of socioeconomic circumstances [19]. For example, parents may have high educational level but still be unemployed, or parents may have low educational level but achieve high occupational status in their work.

Regarding adulthood SES, it seems that high adulthood SES may have a negative influence on compassion for others in adulthood. Current evidence suggests that high adulthood SES is related to weaker abilities to recognize others’ emotional states [19] and less frequent altruistic and helpful behavior toward others [20, 21]. Moreover, cross-sectional neuroimaging studies have found that high adulthood SES is linked to weaker neural responses to empathy-inducing stimuli [22] and weaker activation of the mentalizing and emotional processing networks when observing social situations [23]. Furthermore, high adulthood SES is related to stronger heart rate deceleration in response to other’s suffering, a response characteristic of those with high compassion [24]. Taken together, previous studies suggest that high adulthood SES might have a negative effect on one’s socioemotional development.

However, the link between adulthood SES and compassion for others has remained almost uninvestigated. There exists only one cross-sectional study suggesting that low adulthood SES may correlate with higher dispositional compassion and stronger compassionate states during compassion-inducing stimuli [24]. No longitudinal studies have been conducted.

In the current study, we sought answers to two research questions: (i) does childhood family SES predicts offspring’s compassion for others from early adulthood to middle age (between the ages of 20–50 years), and (ii) does adulthood SES predict the development of compassion in adulthood or vice versa. Indicators of SES included level of income, educational level, occupational status, and employment status. We used population-based data with an intergenerational design and a 32-year prospective follow-up. In our analyses, we took into consideration a variety of potential confounds (age, gender, socioeconomic status in childhood and adulthood, child’s disruptive behavior, parental mental disorder, parents’ frequency of alcohol use and intoxication).

## 2 Materials and methods

### 2.1 Participants

The participants were from the prospective Cardiovascular Risk in Young Finns data. The participants were selected randomly from six age cohorts (born in 1962, 1965, 1968, 1971, 1974, and 1977) who were living in the surrounding municipalities of the Finnish universities with medical schools (i.e. Helsinki, Tampere, Oulu, Kuopio, Turku). The selection was done using the population register of the Social Insurance Institution that covers the whole population of Finland. The original sample included 3596 participants in the baseline measurement in 1980 (when participants were aged 3‒18 years). All the subjects were ethnic Finns. The participants have been followed since then so that the latest follow-up measurement was in 2011 (participants were aged 24‒49 years). The YFS data has been used in numerous previous studies [25–27] and is described with more detail elsewhere [28].

The YFS was approved by all participating universities’ ethics committees at the beginning of the study in 1980, and the follow-ups were approved by the ethics committee of the University of Turku (vernacular institution name: Varsinais-Suomen sairaanhoitopiirin kuntayhtymä, Eettinen toimikunta, Meeting Number 9/2010; study name, “Lasten sepelvaltimotaudin riskitekijät projekti (Laseri) 30-vuotisseurantatutkimus, 25.8.2010”). The study was conducted in accordance with the Helsinki Declaration (WMA Declaration of Helsinki, 2013). Written informed consent was obtained either from the participants or their parents (if the participant was under 18 years old).

The datasets presented in this article are not readily available because YFS is an ongoing follow-up study and the datasets are not anonymised, and the GDPR prevents public sharing of the data. Instead, pseudonymised datasets are possible to share on request, and requires a data sharing agreement between the parties. Requests to access the datasets should be directed to Department of Psychology and Logopedics, University of Helsinki (email katri.raikkonen@helsinki.fi or niklas.ravaja@helsinki.fi).

For the present study, we used data on parental socioeconomic factors in 1980; participants’ socioeconomic factors in 2001 and 2011; and compassion for others in 1997, 2001, and 2012. The measurement years of all the study variables are summarized in S1 Table. In the analyses, we included all the participants who had datable on study variables (e.g. in childhood SES analyses, we included participants with data available on compassion in 1997, 2001, or 2012, on SES in 1980, and data available on covariates). Accordingly, the final sample consisted of 1113–2300 participants in the analyses related to childhood SES (sample size varying in line with number of covariates); and 637–709 participants in the analyses related to adulthood SES (there was more attrition in adulthood vs. childhood SES variables).

### 2.2 Measures

#### 2.2.1 Dispositional compassion

Dispositional compassion for others was evaluated with the version 9 of the Temperament and Character Inventory (TCI) [29]. The compassion scale consists of 10 self-rating statements (e.g., “It gives me pleasure to see my enemies suffer” [reverse scored], “It gives me pleasure to help others, even if they have treated me badly” [positively scored], “I like to imagine my enemies suffering” [reverse scored] and “I hate to see anyone suffer” [positively scored]). The items were answered using a 5-point Likert-scale ranging from 1 (completely disagree) to 5 (completely agree). The score of compassion was calculated for all the participants with data on at least 50% of the items. The scores of compassion in 1997, 2001, and 2012 were standardized with the mean and SD of compassion in 2012, in order to stabilize the growth trajectories of the participants’ levels of compassion over different measurement years.

Confirmatory factor analysis showed that construct validity of the compassion scale was excellent [30]. Additionally, the internal consistency of the scale was found to be high (Cronbach’s *α* = .86–.88 in 1997–2012). The test-retest reliability of the scale has been confirmed previously [31]. Moreover, high values of the compassion scale correlate with higher social warmth toward others, higher altruistic behavior, and higher sociability [32, 33], whereas low values of the compassion scale correlate with higher hostility and aggression toward others [32, 34], higher narcissistic traits [35], and higher psychopathic features [36].

#### 2.2.2 Socioeconomic status (SES)

Socioeconomic status was self-reported by the participants. Participants’ and their parents’ educational level was classified into 3 categories (1 = comprehensive school; 2 = high school or occupational school; 3 = academic level). Participants’ and their parents’ occupational status was defined as manual, lower grade non-manual, or upper grade non-manual. In case mother’s and father’s educational level or occupational status differed from each other, we used the higher value. Level of total parental income included 8 categories (1 = less than 15 000 Finnish mark (2 523€) per year; 8 = more than 100 000 Finnish mark (16 819€) per year). Finally, participants’ and their parents’ employment status was defined as unemployment or not (employment, sick leave, maternal leave etc.). Overall, we had separate indicators of unemployment and occupational status, because unemployment is shown to have vastly different psychological influences depending on previous occupational status at work (see e.g. Paul & Moser, 2009) [37].

The datasets presented in this article are not readily available because YFS is an ongoing follow-up study and the datasets are not anonymised, and the GDPR prevents public sharing of the data. Instead, pseudonymised datasets are possible to share on request, and requires a data sharing agreement between the parties. Requests to access the datasets should be directed to Liisa Keltikangas-Järvinen (liisa.keltikangas-jarvinen@helsinki.fi).

We calculated a SES score for participants (in 2001 and 2011) and their parents (in 1980). For this, all the socioeconomic factors were further classified into 2 categories as follows: educational level (1 = academic level; 0 = lower educational level); occupational status (1 = upper-grade non-manual worker; 0 = lower occupational status); and employment status (1 = neither of parents unemployed; 0 = one parent or both parents unemployed). Participants’ adulthood SES score was calculated as the sum of the 3 dichotomous SES indicators (educational level, occupational status, employment status), ranging between 0–3. Level of income could not be included in participants’ SES score because information about level of income was not available at the baseline measurement. For childhood family SES score, we categorized level of total parental income into two categories (1 = highest 25% in the sample; 0 = lowest 75% in the sample). Childhood family SES score was calculated as the sum of the 4 dichotomous SES indicators (parents’ educational level, occupational status, level of total parental income, employment status), ranging between 0–4. When defining the categories and cut-off values of single SES indicators, we utilized the classification used in a previous study of this same dataset [see 38].

#### 2.2.3 Other covariates of the family environment in childhood

*Parental alcohol use* included the frequency of mother’s and father’s alcohol use (1 = never; 8 = daily) and the frequency of intoxication (1 = never; 8 = daily) that were self-reported by the parents. *Parental mental disorder* was evaluated by asking both parents whether they had ever been diagnosed with any mental disorder (0 = not diagnosed; 1 = diagnosed). *Child’s disruptive behavior* was measured with a questionnaire presented for the parents in 1980. The questionnaire included 6 items that were answered dichotomously (0 = no; 1 = yes). The items were the following: (i) Other children say that my child gets easily into fights. (ii) My child hits/pushes other children "by accident." (iii) My child needs a lot of discipline to control aggression. (iv) My child uses swear words very often. (v) Other children complain often about my child’s behavior. (vi) Other parents have complained about my child’s behavior. In the statistical analyses, the sum score of the items was used.

### 2.3 Statistical analyses

Statistical analyses were conducted using STATA SE version 15.0. Attrition was examined by comparing the included and excluded participants with regard to study variables using chi-square tests and independent samples t-tests. The relationship of parental SES score in childhood with offspring’s compassion in adulthood was investigated using growth curve models (multilevel models for repeated measurements design). In multilevel models, fixed effects refer to the classic regression coefficients, whereas random effects refer to the individual-level variation in the intercept and slopes. We estimated the growth curve for compassion between ages 20 (the age of the youngest age cohort in 1997) to 50 (the age of the oldest age cohort in 2012). Age was centered to the age of 20 years in order to decrease multicollinearity. In model 1 (baseline model), fixed effects were estimated for the parental SES score, age, age-squared, and the interaction effects of parental SES score with age and age-squared. Thus, we estimated both linear and curvilinear associations of the parental SES score with offspring’s compassion over age. In model 2, we added other childhood covariates to the fixed effects (disruptive behavior in childhood; parental mental disorder; frequency of parental alcohol use and alcohol intoxication). In model 3, we added also participants’ SES score in adulthood (education, level of income) to the fixed effects. In all the models, age was treated also as a random effect, in order to consider the individual-level variation in the effect of age on the course of compassion over the follow-up.

The relationship of adulthood SES score with compassion for others was investigated using regression analyses. We conducted two analyses: (1) we predicted compassion in 2012 by adulthood SES score in 2001 (adjusted for age, gender, parental SES score, and baseline compassion in 2001), and (2) we predicted adulthood SES score in 2011 by compassion in 2001 (adjusted for age, gender, parental SES score, and baseline SES score in 2001). In this latter analysis, we used ordinal regression.

## 3 Results

The descriptive statistics of the study variables are shown in Table 1. Attrition analyses revealed that included participants were slightly younger (31.1 vs. 32.1, *p* < .001). Women were more likely to participate than men (69.6% vs. 58.1%, *p* < .001). There was no attrition bias in compassion, childhood family SES score, or adulthood SES score in 2001. However, included participants had slightly higher adulthood SES score in 2011 (1.89 vs. 1.72, *p* = .001) and slightly lower level of disruptive behavior in childhood (0.24 vs. 0.30, *p* < .05). There was no attrition bias in parents’ frequency of alcohol use, parents’ alcohol intoxication, or in the frequency of parental mental disorder.

**Table 1:** The means, standard deviations (SD), frequencies, and ranges of the study variables.

	Mean	SD	Measurement range	Frequency (%)
Age in 2001 (years)	30.09	4.99	24‒39	
Gender (women)				1275 (55.4)
Childhood family SES score (in 1980)	1.90	1.10	0‒4	
Occupational status				
Manual				874 (38.0)
Lower grade non-manual				1000 (43.5)
Upper grade non-manual				426 (18.5)
Educational level				
Comprehensive school				728 (31.7)
High school or occupational school				956 (41.6)
Academic level				616 (26.8)
Level of total parental income (high)				489 (32.8)
Employment status (unemployed)				52 (2.3)
Adulthood SES score (in 2001)	1.34	0.94	0‒3	
Occupational status				
Manual				474 (20.3)
Lower grade non-manual				697 (44.5)
Upper grade non-manual				394 (25.2)
Educational level				
Comprehensive school				129 (6.7)
High school or occupational school				1263 (65.1)
Academic level				549 (28.3)
Employment status (unemployed)				319 (21.8)
Adulthood SES score (in 2011)	1.89	0.88	0‒3	
Occupational status				
Manual				243 (17.7)
Lower grade non-manual				543 (39.4)
Upper grade non-manual				591 (42.9)
Educational level				
Comprehensive school				310 (15.8)
High school or occupational school				1003 (51.1)
Academic level				649 (33.1)
Employment status (unemployed)				75 (5.0)
Disruptive behavior	0.24	0.61	0‒6	
Parental mental disorder				61 (2.7)
Parents’ frequency of alcohol use^1^	4.07	1.81	1‒8	
Parents’ frequency of alcohol intoxication^1^	2.54	1.35	1‒8	
Compassion for others				
1997	3.57	0.70	1‒5	
2001	3.66	0.66	1‒5	
2012	3.74	0.60	1‒5	

^1^ The measurement range: 1 = never; 8 = daily.

### 3.1 Childhood family SES predicting offspring’s compassion in adulthood

**Fig 1:**
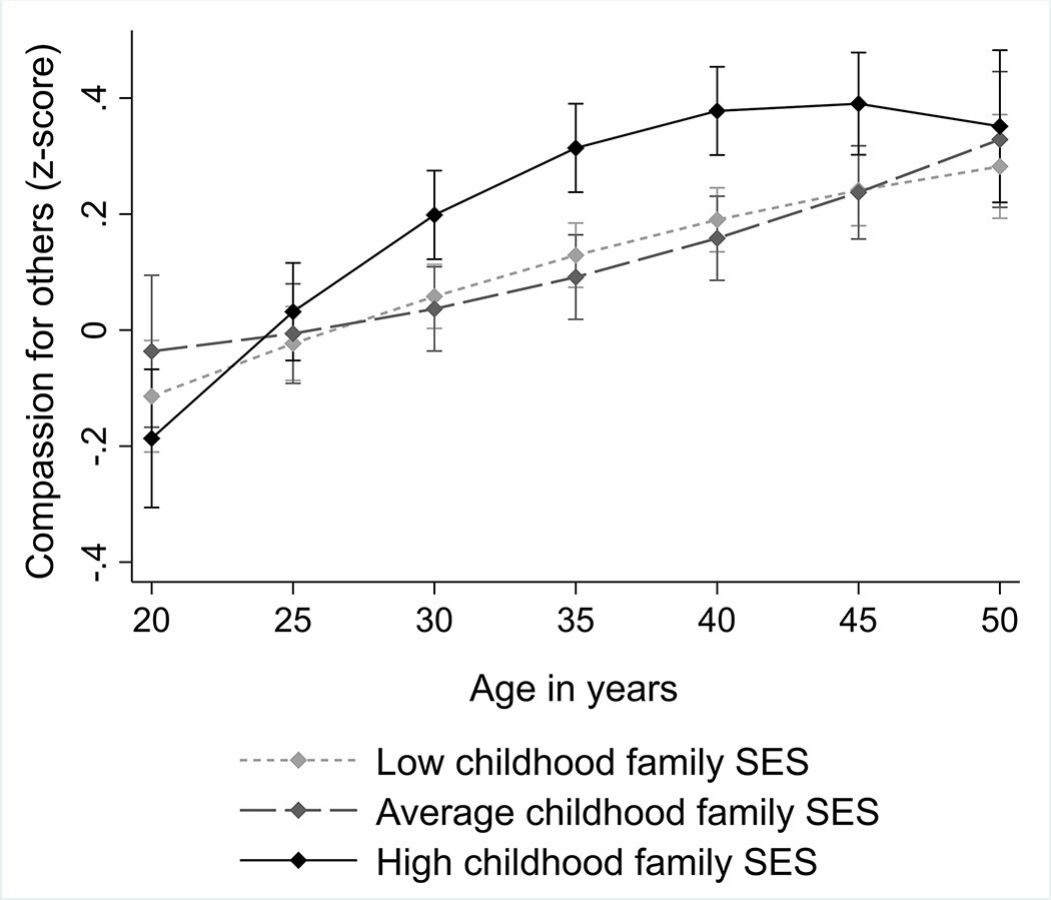
Predicted means with 95% confidence intervals of compassion separately for participants with low (lowest 25% in the sample), average, and high (highest 25% in the sample) parental SES score (*N* = 2300). Adjusted for age and gender.

The results of the multilevel models are shown in Table 2. Childhood family SES did not have any significant main effect on offspring’s compassion in adulthood, expect for the fully-adjusted model where high childhood family SES had a negative main effect on offspring’s compassion. Further, we obtained significant interaction effects of age and age-squared with childhood family SES score. These age-interaction effects were evident in all the models (when adjusted only for age and gender, when adjusted also for childhood covariates, and when adjusted also for participants’ SES score in adulthood). Overall, the results indicated that high childhood family SES predicted offspring’s higher compassion in middle adulthood (approximately at the age of 30–40 years), but not in early adulthood (at the age of 20–30 years) or in middle age (at the age of 45–50 years). The findings are illustrated in Fig 1.

**Table 2:** The results of multilevel models, when predicting the course of compassion by parental SES score.

	Compassion for others					
	Model 1 (*N* = 2300)		Model 2 (*N* = 1994)		Model 3 (*N* = 1113)	
	B	95% CI	B	95% CI	B	95% CI
Fixed effects						
Intercept	-0.211**	-0.348; -0.076	-0.432***	-0.665; -0.199	-0.336*	-0.640; -0.032
Childhood family SES score	-0.029	-0.086; 0.027	-0.042	-0.101; 0.018	-0.075*	-0.150; 0.000
Age	0.00039	-0.015; 0.016	-0.0024	-0.019; 0.015	-0.0075	-0.028; 0.013
Age-squared	0.00034	0.000; 0.001	0.00041	0.000; 0.001	0.00057	0.000; 0.001
Childhood family SES score*Age	0.012**	0.005; 0.019	0.012**	0.005; 0.020	0.012**	0.004; 0.021
Childhood family SES score*Age-squared	0.000**	-0.001; 0.000	-0.00035**	-0.001; 0.000	-0.00037**	-0.001; 0.000
Gender^1^	0.259***	0.190; 0.328	0.230***	0.155; 0.306	0.198***	0.105; 0.292
Disruptive behavior			-0.104**	-0.166; -0.041	-0.062	-0.151; 0.026
Parental mental disorder			0.167	-0.062; 0.396	0.282	-0.049; 0.613
Parents’ frequency of alcohol use			0.021	-0.008; 0.050	0.0074	-0.028; 0.043
Parents’ frequency of alcohol intoxication			-0.062**	-0.099; -0.024	-0.027	-0.073; 0.019
Adulthood SES score					0.111***	0.057; 0.165
Random effects						
Variance of intercept	0.910*	0.861; 0.961	0.899*	0.848; 0.954	0.825*	0.761; 0.894
Variance of age	0.018*	0.012; 0.026	0.017*	0.011; 0.027	0.016*	0.009; 0.028
Residual variance	0.522*	0.504; 0.542	0.522*	0.502; 0.543	0.506*	0.482; 0.531

*** *p* < .001

** *p* < .01

* *p* < .05 ^1^ Male as the reference group.

Model 1: Adjusted for age and gender.

Model 2: Adjusted also for childhood covariates (child’s disruptive behavior, parental mental disorder, parents’ frequency of alcohol use and intoxication).

Model 3: Adjusted also for participants’ SES score in adulthood.

Coefficients (B) with 95% confidence intervals (CI).

As first additional analyses, we reran the analyses so that also depressive symptoms and neuroticism (the mean scores between 1997–2012) were controlled for (N = 1038) (for more details, please see S1 Methods). When predicting compassion development in adulthood, there remained the significant main effect of childhood family SES (B = -0.559, p = 0.005), the age-interaction of childhood family SES (B = 0.032, p = 0.005), and the age-squared-interaction of childhood family SES (B = -0.00043, p = 0.008).

As second additional analyses, we examined the effects of single family SES indicators for offspring’s compassion development (by adding each SES indicator as predictor separately). The results suggested that parents’ occupational status may have most significant association with offspring’s compassion development (when compared to the other SES indicators). However, there were several statistical limitations restricting the interpretation of these additional analyses. Firstly, the frequencies of single indicators were very low (e.g. 52 participants had unemployed parents). Secondly, the measurement ranges of some single indicators were not optimal for growth curve models (e.g. unemployment was a dichotomous indicator). Thirdly, there is evidence for interactions between some SES indicators [37], so that investigating single indicators separately of each other may not be completely justified.

As third additional analyses, we calculated a risk score of childhood family SES, consisting of the sum of low parental educational level (1 = comprehensive school; 0 = higher educational level), low occupational status (1 = manual worker; 0 = higher occupational status); unemployment status (1 = one or both of the parents unemployed; 0 = neither parent unemployed), and low level of total parental income (1 = lowest 25% in the sample; 2 = highest 75% of the sample). Then, we reran the multilevel models using the risk score of childhood family SES. The risk score of childhood family SES did not predict offspring’s compassion in adulthood (see S2 Table).

### 3.2 Adulthood SES score and compassion for others

**Fig 2:**
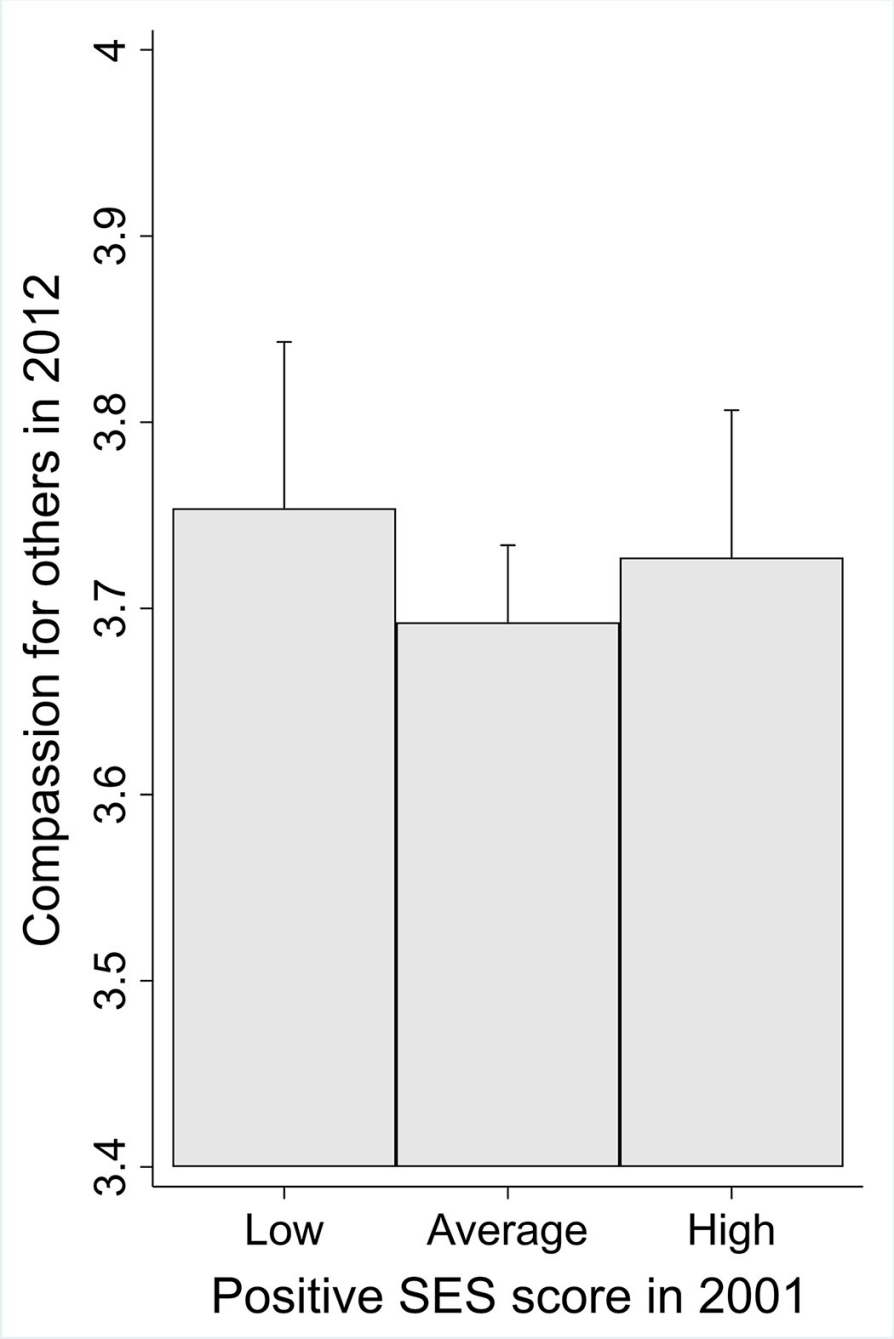
Predicted means with 95% confidence intervals of compassion for others in 2012 with low (lowest 25% in the sample), average, and high (highest 25% in the sample) levels of adulthood SES score (*N* = 709) in 2001. Adjusted for age, gender, parental SES score, and the baseline score of compassion.

**Fig 3:**
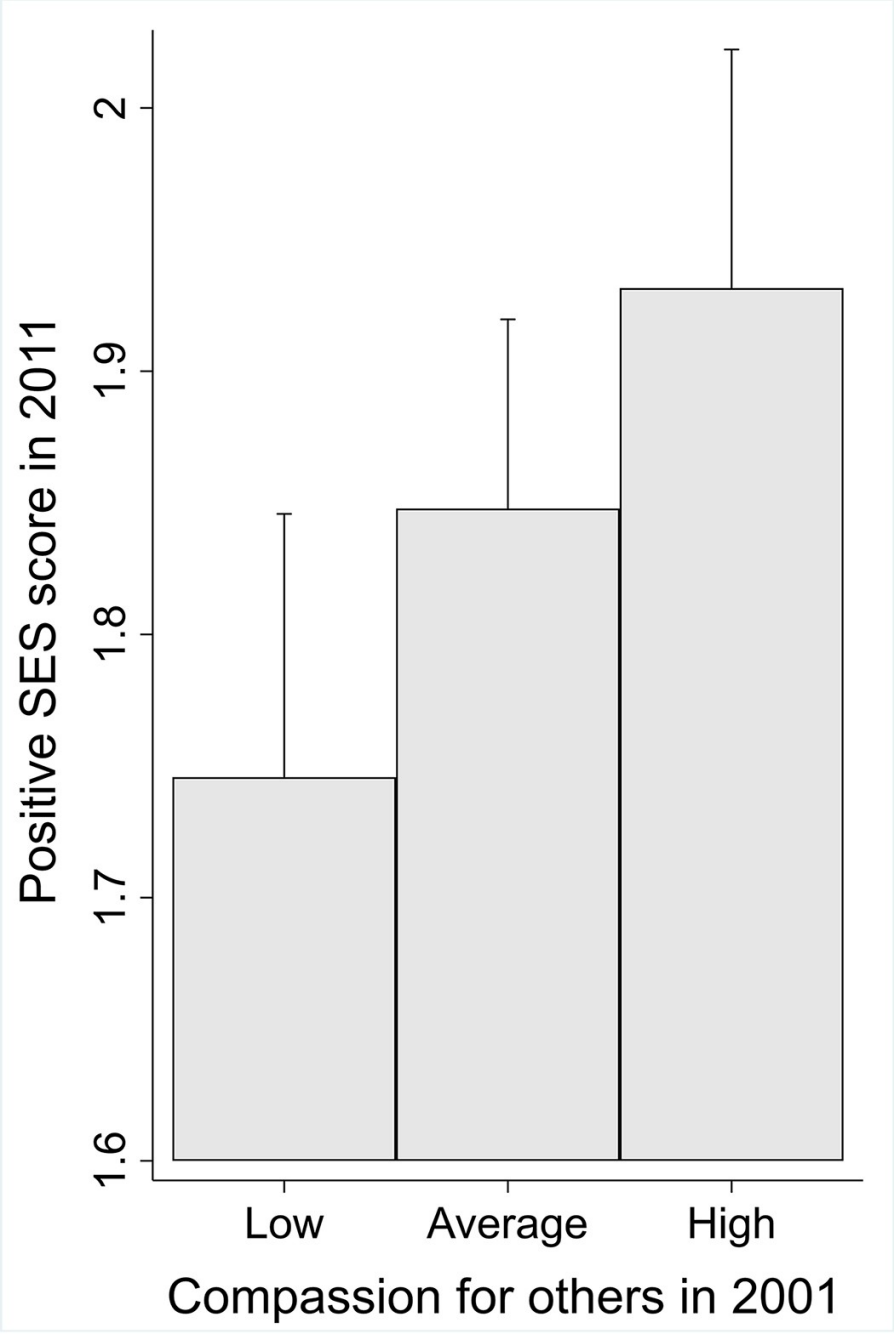
Predicted means with 95% confidence intervals of adulthood SES score in 2011 with low (lowest 25% in the sample), average, and high (highest 25% in the sample) levels of compassion (*N* = 637) in 2001. Adjusted for age, gender, parental SES score, and the baseline adulthood SES score.

The results of the linear regression analyses are presented in Table 3. Adulthood SES did not predict compassion for others over the 10-year prospective follow-up. However, high compassion for others predicted higher adulthood SES score over the follow-up. The findings are illustrated in Figs 2 and 3. These findings were adjusted for age, gender, parental SES score, and the baseline score of adulthood SES (when predicting adulthood SES) or the baseline score of compassion (when predicting compassion).

**Table 3:** The results of regression analyses, when predicting participants’ SES score in adulthood by compassion for others, and vice versa.

	Outcome variable					
	Compassion for others in 2012 (*N* = 709)			Adulthood SES score in 2011 (*N* = 637)		
	B	95% CI	Beta	B	95% CI	OR
Age	0.0086*	0.002; 0.016	0.068	-0.029	-0.063; 0.005	0.971
Gender^1^	0.080*	0.010; 0.151	0.063	0.270	-0.076; 0.616	1.310
Childhood family SES score	0.033*	0.002; 0.064	0.061	0.153	0.000; 0306	1.165
Adulthood SES score in 2001	-0.0017	-0.039; 0.035	-0.003	1.916***	1.666; 2.166	6.792
Compassion for others in 2001	0.616***	0.564; 0.669	0.651	0.296*	0.028; 0.565	1.345

Coefficients (B) with 95% confidence intervals (CI).

* p < .05

** p < .01

*** p < .001

^1^ Male as the reference group.

As first additional analyses, we reran the analyses so that also depressive symptoms (in the baseline measurement in 2001) and neuroticism (in 2007, i.e. in the closest measurement year) were controlled for. All the findings remained. That is, when predicting adulthood SES score in 2011 (N = 569), the significant effect of compassion remained (B = 0.332, p<0.05). When predicting compassion for others in 2012 (N = 650), the effect of adulthood SES score remained non-significant (B = -0.015, p = 0.466), similarly to the main analyses.

As additional analyses, we examined single indicators of SES in adulthood separately (i.e. not as a composite score). The results implied that high compassion may predict lower likelihood for unemployment more strongly than favorable development in other SES indicators (such as level of income or educational level). In contrast, high education may predict higher compassion development more strongly than the other SES indicators. Similarly to childhood SES indicators, however, these additional analyses should be interpreted carefully due to statistical limitations. For example, the frequencies of some SES indicators were very low (e.g. 72 unemployed participants in 2011) and different rates of variance in different SES indicators likely affected the findings.

As third additional analyses, we calculated a risk score of adulthood SES, referring to the sum of low educational level (1 = comprehensive school; 0 = higher educational level), low occupational status (1 = manual worker; 0 = higher occupational status); and unemployment status (1 = unemployed; 0 = not unemployed). Thereafter, we reran all the regression analyses using the risk score of adulthood SES. We obtained no associations between compassion and the risk score of adulthood SES (see S3 Table).

As final additional analysis, we also investigated whether childhood SES modifies the effect of adulthood SES on compassion development. That is, we added the interaction effect between childhood SES and adulthood SES to the model when predicting the course of compassion. The interaction effect was not significant.

## 4 Discussion

This study showed that high childhood family SES predicts offspring’s higher compassion in middle adulthood (approximately at the age of 30–40 years), but not in early adulthood or middle age. Hence, living in economically advantaged circumstances seemed to have a positive influence on one’s disposition to feel compassion for others in adulthood. Moreover, we found that high compassion in adulthood predicted higher adulthood SES, but not vice versa.

The positive relationship of childhood family SES with offspring’s compassion is in line with previous literature. Previous studies suggest that high childhood family SES is strongly linked to favorable psychosocial qualities of home environment that may promote compassion development. Specifically, high family SES is proposed to be linked to lower parental stress levels [12, 14], higher maternal support for the offspring [12, 14], higher quality of the parent-child communication [16], and better family climate [12]. High quality of the parent-child relationship, in turn, predicts offspring’s higher compassion in adulthood [39].

Importantly, the influence of childhood family SES on offspring’s compassion was not significant in early adulthood (approximately ages of 20–25 years) or in middle age (approximately ages of 45–50 years). This study did not investigate potential mechanisms between SES and compassion but there may be some potential explanations. Firstly, it may be that compassion-related qualities need to be a comparatively stable feature of one’s identity, before one is able to conduct prosocial actions toward outgroup-individuals and to forgive for others who have behaved aggressively [40]. In order to form a stable identity, one needs to mentally go through the past events in childhood and adolescence, including parenting practices and childhood family circumstances. Previously, the age of 30 years is found to be a critical age period for personality traits to become more stabilized [41]. We speculate that this may potentially provide one explanation why parental SES predicted compassion beginning at the age of 30 years. Secondly, compassion increased over age in all the SES groups. Hence, in middle age, there may have not been enough variance in compassion to obtain statistically significant differences between SES groups. Thirdly, there were fewer participants in extreme age ranges that may likely resulted in broader confidence intervals and weaker statistical significance of the associations.

The results showed that high compassion predicts higher SES in adulthood. This may be explained by the motivational component of compassion leading to higher willingness to prosocial behavior [3] that, in turn, is related to higher social connectedness [42]. Further, experiencing compassion is related to more frequent actions to promote common goals in one’s social communities [43]. These social benefits of compassion may promote compassionate individuals’ higher status at occupational environments. In addition, compassion may protect against work stress and burnout because high compassion is related to better coping with stress [44, 45] and more favorable health behavior [46].

Previous studies have suggested that high adulthood SES is related to weaker compassion-related qualities, such as lower ability to recognize others’ emotional states [19] and less frequent altruistic and helpful behavior toward others [20, 21]. Those studies, however, did not control for childhood family SES. In this study, we took into account childhood family SES and obtained no association between offspring’s adulthood SES and compassion.

The current study had some methodological limitations that are necessary to be taken into consideration. In Finland, there is a comprehensive social welfare system with quite a strong progressive taxation. Further, unemployed individuals are typically provided with satisfactory unemployment benefits. Additionally, there is a 9-year-long comprehensive school for the whole age group, so that all the citizens may likely have basic educational knowledge. Hence, even “low SES” may likely refer to satisfactory levels of socioeconomic circumstances (i.e. having apartment, food, and health care) and, conversely, there are very few individuals with extreme wealth in Finland. Consequently, our results cannot be generalized to populations with extremely low and high levels of SES where the link between compassion and SES might be different. In addition, there may be possible cultural differences in the SES-compassion relationships that may restrict the generalizability of our findings and that could be addressed in up-coming studies. Overall, our findings suggest that even comparatively small increases in childhood family SES (within a reasonable SES range) have a beneficial influence on offspring’s compassion in adulthood.

This study had also a variety of strengths. Firstly, to our knowledge, this study was the first to investigate the relationship of childhood family SES with compassion over a long-term prospective follow-up (32 years) into adulthood. Further, we investigated the relationship between adulthood SES and compassion over an 11-year follow-up. Secondly, we could take into consideration a variety of other covariates (child’s disruptive behavior, parental mental disorder, parents’ frequency of alcohol use and intoxication). Thirdly, we used SES composite scores consisting of several SES indicators (level of income, occupational status, educational level, employment status), in order to capture the multidimensional aspects of socioeconomic circumstances. Fourthly, we had a large population-based sample with intergenerational design and three respondents from each family (mother, father, and child). Finally, as academic-level education is provided free-or-charge for the Finnish citizens, childhood family SES may not largely determine offspring’s SES development. Hence, the effects of one’s own characteristics (such as compassion) on later SES development can be more clearly observed in our Finnish sample than in some other countries.

Commonly, unemployment or other socioeconomic troubles are treated using public employment services such as vocational training [47]. There is evidence, however, that compassion may have favorable influences on socioeconomic status: for example, compassionate practices at work place are found to predict higher work engagement and to protect against burnout in stressful circumstances over a 6-month follow-up [48]. Our study showed that compassion is related to higher socioeconomic status over an 11-year follow-up. Further, there is evidence that compassion may be enhanced even with a few-week-long compassion intervention [49], including practices to e.g. increase tolerance to other’s suffering and to shift attention from self-monitoring to recognizing others’ emotional states [50]. Finally, there is evidence that women coming from low-SES childhood families may not be willing to contact health-care professionals and must be contacted even ten times in order to get them to participate in psychotherapy [51]. Our study suggests that individuals with low childhood SES may have a lower level of compassion that, in turn, may potentially be manifested as a distrust toward health-care professionals. Consequently, individuals coming from socioeconomically harsh environments could be treated with particular warmth and trust, as has been suggested also previously [52].

## Supporting information

S1 Table The measurement years of the study variables. (DOCX)

S2 Table The results of multilevel models, when predicting the course of compassion by parental SES risk score. Coefficients (B) with 95% confidence intervals (CI).
(DOCX)

S3 Table The results of regression analyses, when predicting participants’ SES risk score in adulthood by compassion for others, and vice versa. Coefficients (B) with 95% confidence intervals (CI).
(DOCX)

S1 Methods A description of the measures of neuroticism and depressive symptoms that were used in the additional analyses. (DOCX)
