# Supplementary material for: The relationship of socioeconomic status in childhood and adulthood with compassion: A study with a prospective 32-year follow-up
Source: PLoS One. 2021 Mar 24;16(3):e0248226. doi: 10.1371/journal.pone.0248226 (PMC7990193; doi:10.1371/journal.pone.0248226)
Supplement: S1 Table — (DOCX) [file pone.0248226.s001.docx]

**S1 Table.**

|  | 1980 | 1997 | 2001 | 2007 | 2011/2012 |
| --- | --- | --- | --- | --- | --- |
| Compassion for others |  | X | X |  | X |
| Childhood family SES score | X |  |  |  |  |
| Adulthood SES score |  |  | X |  | X |
| Other childhood covariates |  |  |  |  |  |
| Child’s disruptive behavior | X |  |  |  |  |
| Parental mental disorder | X |  |  |  |  |
| Parents’ frequency of alcohol use | X |  |  |  |  |
| Parents’ frequency of alcohol intoxication | X |  |  |  |  |
