# Supplementary material for: The relationship of socioeconomic status in childhood and adulthood with compassion: A study with a prospective 32-year follow-up
Source: PLoS One. 2021 Mar 24;16(3):e0248226. doi: 10.1371/journal.pone.0248226 (PMC7990193; doi:10.1371/journal.pone.0248226)
Supplement: S2 Table — Coefficients (B) with 95% confidence intervals (CI). (DOCX) [file pone.0248226.s002.docx]

**S2 Table.**

|  | Compassion for others | | | | | | | |
| --- | --- | --- | --- | --- | --- | --- | --- | --- |
|  | Model 1 (*N*=2300) | |  | Model 2 (*N*=1994) | |  | Model 3 (*N*=1113) | |
|  | B | 95% CI |  | B | 95% CI |  | B | 95% CI |
| Fixed effects |  |  |  |  |  |  |  |  |
| Intercept | -0.244*** | -0.336; -0.153 |  | -0.146* | -0.282; -0.010 |  | -0.031 | -0.204; 0.141 |
| Childhood family SES risk score | -0.036 | -0.103; 0.031 |  | -0.022 | -0.095; 0.051 |  | -0.010 | -0.105; 0.085 |
| Age | 0.027*** | 0.016; 0.037 |  | 0.026*** | 0.015; 0.037 |  | 0.023*** | 0.010; 0.035 |
| Age-squared | -0.00040* | -0.001; -0.000 |  | -0.00042* | -0.001; -0.000 |  | -0.00038 | -0.001; 0.000 |
| Childhood family SES risk score*Age | -0.002 | -0.010; 0.006 |  | -0.003 | -0.012; 0.006 |  | -0.0048 | -0.016; 0.006 |
| Childhood family SES risk score*Age-squared | 0.000076 | -0.000; 0.000 |  | 0.00012 | -0.000; 0.000 |  | 0.00020 | -0.000; 0.001 |
| Gender^1^ | 0.258*** | 0.188; 0.327 |  | 0.229*** | 0.153; 0.305 |  | 0.151** | 0.053; 0.248 |
| Disruptive behavior |  |  |  | -0.107** | -0.169; -0.045 |  | -0.074 | -0.163; 0.014 |
| Parental mental disorder |  |  |  | 0.162 | -0.068; 0.392 |  | 0.314 | -0.019; 0.647 |
| Parents' frequency of alcohol use |  |  |  | 0.026 | -0.003; 0.054 |  | 0.008 | -0.027; 0.044 |
| Parents' frequency of alcohol intoxication |  |  |  | -0.066*** | -0.103; -0.029 |  | -0.031 | -0.077; 0.015 |
| Adulthood SES risk score |  |  |  |  |  |  | -0.165*** | -0.284; -0.046 |
|  |  |  |  |  |  |  |  |  |
| Random effects |  |  |  |  |  |  |  |  |
| Variance of intercept | 0.907* | 0.858; 0.958 |  | 0.896* | 0.845; 0.951 |  | 0.823* | 0.759; 0.892 |
| Variance of age | 0.018* | 0.012; 0.026 |  | 0.017* | 0.011; 0.027 |  | 0.016* | 0.009; 0.028 |
| Residual variance | 0.523* | 0.505; 0.543 |  | 0.524* | 0.503; 0.545 |  | 0.508* | 0.484; 0.533 |
| *** *p*<.001 ** *p*<.01 * *p*<.05 ^1^ Male as the reference group. | | | | | | | | |
| Model 1: Adjusted for age and gender. | | | | | | | | |
| Model 2: Adjusted also for childhood covariates (child’s disruptive behavior, parental mental disorder, parents’ frequency of alcohol use and intoxication). | | | | | | | | |
| Model 3: Adjusted also for participants’ SES risk score in adulthood. | | | | | | | | |
