# Supplementary material for: The relationship of socioeconomic status in childhood and adulthood with compassion: A study with a prospective 32-year follow-up
Source: PLoS One. 2021 Mar 24;16(3):e0248226. doi: 10.1371/journal.pone.0248226 (PMC7990193; doi:10.1371/journal.pone.0248226)
Supplement: S3 Table — Coefficients (B) with 95% confidence intervals (CI). (DOCX) [file pone.0248226.s003.docx]

**S3 Table.**

|  | Outcome variable | | | | | | |
| --- | --- | --- | --- | --- | --- | --- | --- |
|  | Compassion for others in 2012  (*N*=709) | | |  | Adulthood SES risk score in 2011  (*N*=637) | | |
|  | B | 95% CI | Beta |  | B | 95% CI | Beta |
| Age | 0.009* | 0.002; 0.016 | 0.069 |  | 0.003 | -0.003; 0.009 | 0.026 |
| Gender^1^ | 0.079* | 0.008; 0.149 | 0.062 |  | -0.260*** | 0.206; 0.295 | -0.299 |
| Childhood family SES risk score | -0.012 | -0.045; 0.022 | -0.019 |  | 0.029* | 0.001; 0.058 | 0.070 |
| Adulthood SES risk score in 2001 | 0.031 | -0.020; 0.081 | 0.035 |  | 0.250*** | 0.206; 0.295 | 0.386 |
| Compassion for others in 2001 | 0.620*** | 0.567; 0.673 | 0.655 |  | -0.008 | -0.053; 0.037 | -0.012 |
| * p<.05 ** p<.01 *** p<.001  ^1^ Male as the reference group. | | | | | | | |
