# Supplementary material for: The relationship of socioeconomic status in childhood and adulthood with compassion: A study with a prospective 32-year follow-up
Source: PLoS One. 2021 Mar 24;16(3):e0248226. doi: 10.1371/journal.pone.0248226 (PMC7990193; doi:10.1371/journal.pone.0248226)
Supplement: S1 Methods — (DOCX) [file pone.0248226.s004.docx]

**S1 Methods.** A description of the measures of neuroticism and depressive symptoms that were used in the additional analyses.

*Neuroticism.* Neuroticism was measured with the NEO-FFI self-report questionnaire in 2007 and 2012. The subscale of Neuroticism included 12 items that were rated with a 5-point scale (1=totally disagree; 5=totally agree) (Cronbach’s α =0.89). For each measurement year, we calculated the mean score of the items (higher scores referred to higher neuroticism) for all the participants with at most 50% missing items.

*Depressive symptoms.* Depressive symptoms were measured with a modified version of the Beck Depression inventory (mBDI) in 1997, 2001, and 2012. The mBDI consists of the second mildest statements of the BDI (i.e. the items that represented the second mildest symptoms in the original BDI). The items were rated with a 5-point scale (1=totally disagree; 5= totally agree) (Cronbach’s α=0.91−0.91 in 1997, 2001, and 2012). A more detailed description of the mBDI is available elsewhere (Elovainio et al., 2005, Journal of Psychiatric Research). Compared to the original BDI, the mBDI is noted to capture subclinical depressive symptoms of a population-based sample more sensitively (e.g.  Rosenström et al., 2012, Plos One). For each measurement year, we calculated a mean score of the items (higher scores referred to higher depressive symptoms) for all the participants who had data available on at least 50% of the items.
